# Post-Bariatric Surgery Patients: A Quality of Life Assessment in Saudi Arabia

**DOI:** 10.7759/cureus.24273

**Published:** 2022-04-19

**Authors:** Abdullah A Alotaibi, Ahmad N Almutairy, Anas S Alsaab

**Affiliations:** 1 Family Medicine, King Abdulaziz Medical City Riyadh, Riyadh, SAU; 2 Family Medicine, King Saud Medical City, Riyadh, SAU

**Keywords:** medicine, surgery, obesity, quality of life, bariatric surgery

## Abstract

Background

Bariatric surgery has emerged as the most successful and long-lasting treatment for weight loss because of its effect on losing weight, diminution or elimination of sedentary lifestyle-related comorbidities, and a concomitant decrease in mortality and healthcare expenditures. Understanding its effects on the quality of life of patients is a crucial aspect of treatment. This study aims to assess the quality of life and its associated factors following bariatric surgery in Saudi Arabia.

Methodology

In this cross-sectional study, 400 adult patients who had undergone bariatric surgery were recruited from different regions in Saudi Arabia between December 2021 and February 2022. Data were collected and analyzed using SPSS version 23 (IBM Corp., Armonk, NY, USA).

Results

Of the 400 participants, 160 (40%) were males and 240 (60%) were females. The average weight of the participants before surgery was 113.9 ± 18.7 kg (range = 84-146 kg) and after surgery was 84.8 ± 18.3 kg (range = 58-138 kg). The average quality of life score was -0.13 ± 2.0 (range = -3-3). Most of the participants (35%) had a fair quality of life, 25% had very poor quality, 20% had very good quality, 15% had good quality, and 5% had poor quality of life. A statistically significant association (p < 0.001) was found with the following factors: gender, age, nationality, educational level, marital status, and history of chronic diseases.

Conclusions

Quality of life following bariatric surgery was mostly reported to be fair or average. Several sociodemographic factors were found to be associated with the reported quality of life including age, gender, nationality, educational level, marital status, and history of chronic disease or comorbidities.

## Introduction

Bariatric surgery, also known as weight loss surgery, refers to a range of procedures done on obese individuals [1]. Long-term weight loss is achieved mostly through quality-of-care procedures such as adjusting intestinal levels of hunger and satisfaction chemicals and achieving a new biological body mass index (BMI) setpoint value [2]. Long-term trials have shown that the treatments lead to massive weight loss, insulin restoration, reductions in cardiovascular risk, and a 40-23% reduction in fatalities. According to the National Institutes of Health, obese adults with a BMI of at least 40 and those with a BMI of at least 35 and major comorbid medical disorders such as diabetes should consider bariatric surgery [3]. However, new data indicate that bariatric surgery may be suitable for individuals with a BMI of 35-40 and no illnesses and those with a BMI of 30-35 with substantial comorbid conditions. According to the most recent assessment, individuals with a BMI of more than 30 and comorbidities are suitable candidates for bariatric surgery [4].

In the United States, three main weight loss treatments are currently used on a regular basis, namely, Roux-en-Y gastric bypass (RYGB), variable gastric banding, and sleeve gastrectomy (SG). These procedures have both benefits and drawbacks, and none of them are a quick or easy way of losing weight. In 2013, RYGB was the most frequently performed operation in the world, representing 45% of all procedures, followed by SG (37%) and adjustable gastric banding (10%). From 2003 to 2008, the number of RYGBs performed reduced, further reducing from 2011 to 2013. From 2003 to 2013, the number of SG procedures performed increased dramatically, making it the second most common bariatric operation globally. Adjustable gastric banding (AGB) peaked in 2008, and then declined significantly in 2011 and 2013. Biliopancreatic diversion with duodenal switch also decreased from 1.7% in 2003 to 1.5% in 2013 [5].

With substantial weight loss, diminution or elimination of sedentary lifestyle-related comorbidities, and a concomitant decrease in mortality and healthcare expenditures, bariatric surgery has emerged as the most successful and long-lasting possible treatment for weight loss. These are objective and quantifiable results that lend themselves to analysis [6]. Weight loss after bariatric surgery is associated with a decline in weight gain disorders, such as diabetes, metabolic disease, and sleep issues, but its effect on hypertension is uncertain. Regarding managing comorbidities, it is uncertain whether one bariatric operation is better than another. There are no high-quality data on the long-term effects of comorbidities compared to standard therapeutic interventions and the related benefits and risks of gastric surgery, which has a high burden of diabetes recurrence. There appears to be a significant interest in providing this treatment to patients with type 2 diabetes who have a lower BMI than is generally required for bariatric surgery [7]. Increased evidence, on the other hand, is equivocal, and the ideal timing for surgery is uncertain [7].

When compared with other obesity therapies, weight reduction surgery in adults is associated with many complications [8]. Weight loss surgery has a 4% chance of causing severe problems. Of the three techniques, SG has been shown to be associated with the lowest morbidity and revascularization rates. As per a 2012 research by the American Society for Metabolic and Bariatric Surgery, the proportion of surgeries requiring reoperations due to complications was 15.3% for the gastric band, 7.7% for gastric bypass, and 1.5% for SG [9]. There is little information available regarding its impressive accomplishment and related illnesses when considering the early onset of obesity, years of being overweight, preoperative BMI, Edmonton Obesity Staging System (EOSS) score, and age as potential predictors of weight loss, a maximum aperture of comorbidities, and the associated complications [10]. Markus et al. reported that some post-bariatric patients have high reliability three years after their initial treatment, as well as the quality of life scores, compared to the general population [11].

Quality of life in terms of healthy life is a multidimensional concept that captures physiological, emotional, and social elements of wellness [12]. It is a multifaceted concept of an individual’s perspective of the detrimental effect of an illness. Severe obesity, characterized by high BMI in the presence of adiposity disease [13], is linked to numerous comorbidities, a shorter lifespan, and a lower health-related quality of life (HRQoL). The need for a better quality of life is a primary motivator for considering bariatric surgery [14,15]. Moreover, bariatric surgery patients preoperatively are at an exceptionally high risk of having a lower HRQoL among patients with morbid obesity [16].

In a previously reported study, HRQoL preoperatively and five years after bariatric surgery were found through a comprehensive database search. The outcome of interest was the improvement in HRQoL over time, separated into direct and indirect events. Seven prospective studies met the selection criteria. There were eight HRQoL assessments and six surgical techniques. Lengthy follow-up lasted 5-10 years, with sample sizes ranging from 44 to 655 individuals, and carry rates ranging from 61% to 92%. HRQoL outcomes were frequently noted during the initial years of follow-up, with a progressive decline that appeared to stabilize five years later. Long-term HRQoL levels were usually much better than facilitating greater but still fell short of normative data. Applicants for bariatric surgery had poor HRQoL before surgery, but their HRQoL improved significantly after surgery, and most of the early HRQoL gains were sustained over time [17].

Another study included 34 patients who had undergone laparoscopic SG and 31 who had undergone laparoscopic RYGB. Before the operation, the mean weight was 146.2 kg. Before the surgical procedure, 89% of those eligible for the surgical treatments had hypertension, and 52% had type 2 diabetes. Before beginning the surgical therapy, the quality of life was examined. The quality of life was significantly low in both groups that qualified for bariatric surgeries. The standard of living of patients improved after the treatments. The total intensity of concomitant diseases also reduced dramatically during a follow-up visit. Type 2 diabetes and hypertension improved in 26 of 34 patients and 42 of 58 patients, respectively [18]. According to the study by Coupaye et al., loss of body mass one year after SG and one year after a mini gastric bypass was 35.9 kg and 38.6 kg, respectively. In this case, too, the ultimate outcomes in both groups were comparable [19].

Obesity has been linked with a higher risk of type 2 diabetes mellitus, cholesterol, hypertension, airflow obstruction syndrome, gastroesophageal reflux disease (GERD), and reduced quality of life. Obesity also affects both personal and social performance [20]. It has become one of the most severe hazards to public health in the 21st century. Since 1980, the global incidence has doubled, with an average of 12-13% having a BMI of 30 kg/m^2^ or higher [21,22].

A study was conducted to analyze the differences in the standard of living for the two commonly prevalent procedures, namely, laparoscopic SG and laparoscopic gastric bypass. Patients who received SG or RYGB as a planning control between January 2012 and January 2017 were included if they filled the quality of life questionnaire both preoperatively and one year thereafter. Quality of life was measured using the RAND36 health survey [13]. Regarding excessive weight loss, hypertension, dyslipidemia, GERD, and osteoarthritis, a meta-analysis of 62 recent trials found that RYGB outperformed SG in terms of excess weight loss, hypertension, dyslipidemia, GERD, and arthritis. On type 2 diabetes mellitus and obstructive sleep apnea, the results were comparable [23].

In another study, between January 2012 to January 2017, 3,768 patients were operated on. Revision operations, bandage removals, and one-anastomosis bypasses were all ruled out first. After ruling out these operations, a total of 1,184 patients were included in the analysis. In total, 666 patients received SG, and 535 patients received RYGB. Before the procedure, patients tended to score worse in all domains. Patients who underwent RYGB experienced a considerable level of physical function. This study was consistent with previous work that bariatric surgery increases HRQoL across the board. Patients who underwent RYGB reported a considerably higher effect on physical performance and fewer GERD symptoms [15].

Another study looked at the psychological predictors of psychiatric conditions and the quality of life after bariatric surgery. Documents with a random sample of 30 or more and at least a year of compliance were included in the study conducted between 2003 and 2012. According to the research, preoperative psychological characteristics, such as psychiatric disorders, body issues, and ego, may be essential for postpartum mental health. Personality, severe psychiatric disease at the start, and remission of depressed symptoms appeared to predict postoperative HRQoL. Furthermore, postoperative mental problems and incorrect eating behavior were likely to lead to a poor health standard of living outcome [24].

## Materials and methods

A cross-sectional study was conducted in different regions of Saudi Arabia from December 2021 to February 2022. We included post-bariatric patients regardless of the type of surgery. The inclusion criteria were any adult Saudi Arabian residents who underwent bariatric surgery and agreed to participate in the study, of both genders, of any nationality, could read, and had a social media account. Non-Saudi Arabian residents, with no history of bariatric surgery, no social media account, and who refused to participate in the study were excluded from the study population. The sample size was calculated using the EPI info program. Based on a 95% confidence interval (CI), 5% margin of error, and the total population of post-bariatric patients in Saudi Arabia, the estimated sample size was 384, which was adjusted to 422 to compensate for a 10% non-response rate. The study was conducted using an online self-administered questionnaire distributed via Google Forms. The generated link was conveniently shared on social media (i.e., Facebook, WhatsApp, Telegram, and Twitter). The aim of the study was clearly explained on the interface.

A validated questionnaire was used based on previous studies from Moorehead et al. (Moorehead-Ardelt Quality of Life Questionnaire II (M-A QoLQII) Scoring Key). The questionnaire included questions on the sociodemographic characteristics of the participants such as age group, sex, and nationality. Written informed consent was obtained from all participants. The questionnaire also included questions about the type of the surgery, time of the surgery, background medical conditions, and, finally, quality of life assessment. A common scoring system based on the validated questionnaire was used (very poor: -3 to -2.1; poor: -2 to -1.1; fair: -1 to 1; good: 1.1 to 2; very good: 2.1 to 3).

The questionnaire was pretested in a pilot study in a sample of 20 participants whose results were not included in this study. Some modifications were done accordingly to ensure clarity and easy understanding of the questions. A convenient non-probability sampling technique was used to select the participants. Data were coded, entered, and analyzed using SPSS version 23 (IBM Corp., Armonk, USA). Qualitative data were expressed in the form of numbers and percentages. Chi-square (χ2) test was used to examine qualitative data between two groups.

This study was approved by the Research Ethics Committee of King Saud Medical City (reference number: H1RI-01-Dec21-01). All data were kept confidential and used only for research purposes.

## Results

Characteristics of the study participants

A total of 400 (94.8%) participants completed the questionnaires. Of the 400 participants, 160 (40%) were males and 240 (60%) were females. The age groups of the participants were as follows: 16 to 25 years (25%), 26 to 35 years (25%), 36 to 45 years (45%), and 46 to 65 years (5%). The majority of the participants were Saudi Arabian nationals (75%). Overall, 40% of the participants completed their bachelor’s degree and 40% were completing their secondary school. The majority of the participants were married (65%) while the remaining were single (35%). Regarding their history of chronic illnesses, 100 (25%) had hypothyroidism, 40 (10%) had hypertension, 20 (5%) had dyslipidemia, 20 (5%) had infertility, and 60 (15%) had other illnesses, while 240 (60%) had no chronic diseases. The characteristics of the participants are summarized in Table 1.

**Table 1:** Characteristics of the participants (n = 400).

Variable	Category	Frequency	Percentage
Gender	Male	160	40%
	Female	240	60%
Age (years)	16–25	100	25%
	26–35	100	25%
	36–45	180	45%
	46–65	20	5%
Nationality	Saudi	300	75%
	Non-Saudi	100	25%
Educational level	Primary	0	0%
	Intermediate	60	15%
	Secondary	160	40%
	Bachelor	160	40%
	Other	20	5%
Marital status	Single	140	35%
	Married	260	65%
	Divorced	0	0%
	Widow	0	0%
History of chronic disease	Yes	160	40%
	No	240	60%

Bariatric surgery information

The majority of the participants underwent sleeve surgery (80%), 5% underwent other surgeries, and 15% did not know what type of surgery they underwent. More than half of the participants (55%) had surgery less than six months ago. The average weight of the participants before surgery was 113.9 ± 18.7 kg (range = 84-146 kg), while the average weight after surgery was 84.8 ± 18.3 kg (range = 58-138 kg); the decrease in weight after surgery was statistically significant (p < 0.01). Before surgery, most participants had a BMI greater than 30 (65%) while the others had a BMI between 18 and 25 (10%) or did not know their BMI (25%). After the surgery, most participants did not know their BMI (55%) while the others had a BMI between 26 and 30 (20%), greater than 30 (15%), or between 18 and 25 (10%) (Table 2).

**Table 2:** Bariatric surgery information and changes in weight and BMI. BMI: body mass index

Question	N	%
What is the type of the surgery?		
Sleeve surgery	320	80%
Others	20	5%
I don’t know	60	15%
When did you have the surgery?		
<6 months	220	55%
6–12 months	80	20%
1–3 years	40	10%
>3 years	60	15%
What was your BMI before the surgery?		
Below 18	0	0
Between 18 and 25	40	10%
Between 26 and 30	0	0
Above 30	260	65%
I don’t know	100	25%
What is your BMI now?		
Below 18	0	0
Between 18 and 25	40	10%
Between 26 and 30	80	20%
Above 30	60	15%
I don’t know	220	55%

Quality of life assessment of post-bariatric surgery patients and its associated factors

To assess the quality of life of post-bariatric surgery patients a validated questionnaire was used based on previous studies from Moorehead et al. (M-A QoLQII Scoring Key). The average score was -0.13 ± 2.0 (range = -3 to 3). Most participants (35%) had a fair quality of life, 25% had very poor quality, 20% had very good quality, 15% had good quality, and 5% had poor quality of life (Table 3).

**Table 3:** Quality of life assessment.

Quality of life categories	N	%
Very poor (-3 to -2.1)	100	25%
Poor (-2 to -1.1)	20	5%
Fair (-1 to 1)	140	35%
Good (1.1 to 2)	60	15%
Very good (2.1 to 3)	80	20%

We conducted the chi-square test to explore the relationship between the quality of life of the participants and different sociodemographic factors. A statistically significant association (p < 0.001) was found with the following factors: gender, age, nationality, educational level, marital status, and history of chronic disease. Overall, the following characteristic was observed among those who were considered as having good/very good quality of life: males (50%), 36 to 45-year age group (44.4%), Saudi nationals (46.7%), other degree holders (100%), married (38%), and participants with no history of chronic disease (50%). More information about the factors associated with quality of life of post-bariatric surgery patients is presented in Table 4.

**Table 4:** Factors associated with quality of life of post-bariatric surgery patients.

Variable	Categories	Quality of life			P-value
		Poor/Very poor	Fair	Good/Very good	
Gender	Male	60 (37.5)	20 (12.5)	80 (50)	<0.001
	Female	60 (25)	120 (50)	60 (25)	
Age (years)	16–25	60 (60)	20 (20)	20 (20)	<0.001
	26–35	0 (0)	60 (60)	40 (40)	
	36–45	40 (22.2)	60 (33.3)	80 (44.4)	
	46–65	20 (100)	0 (0)	0 (0)	
Nationality	Saudi	80 (26.7)	80 (26.7)	140 (46.7)	<0.001
	Non-Saudi	40 (40)	60 (60)	0 (0)	
Educational level	Intermediate	0 (0)	60 (100)	0 (0)	<0.001
	Secondary	60 (37.5)	60 (37.5)	40 (25)	
	Bachelor	60 (37.5)	20 (12.5)	80 (50)	
	Other	0 (0)	0 (0)	20 (100)	
Marital status	Single	60 (42.9)	40 (28.6)	40 (28.6)	<0.001
	Married	60 (23.1)	100 (38.5)	100 (38.5)	
History of chronic disease	Yes	80 (50)	60 (37.5)	20 (12.5)	<0.001
	No	40 (16.7)	80 (33.3)	120 (50)	

## Discussion

The increasing prevalence of obesity among the Saudi population has led to an increase in the number of bariatric surgeries in the past 20 years, representing an effective and reliable method for weight loss and decreasing obesity-related complications [25]. There are no available data regarding the assessment of the quality of life and its associated factors following bariatric surgery in Arab countries.

This study aimed to assess the quality of life post-bariatric surgery in Saudi Arabia using a validated questionnaire based on previous studies from Moorehead et al. (M-A QoLQII Scoring Key). The distribution of age and gender was similar to that reported by previous studies conducted among our population of interest [25,26].

In this study, most participants (35%) had a fair quality of life, 25% had very poor quality, 20% had very good quality, 15% had good quality, and 5% had poor quality of life compared to another study conducted in the United States using the same questionnaire, where most participants (36%) had a good quality of life, 12% had poor quality of life, 19% had a fair quality of life, and 32% had a very good or excellent quality of life, which represents a better quality of life post-bariatric surgery than that reported in our study [27].

This difference in the reported quality of life might be due to the different types of bariatric procedures in the two studies. The US-based study was conducted among patients after laparoscopic AGB [26], whereas in our study the most prevalent type of surgery (80%) was sleeve surgery.

Comparing different types of bariatric procedures is one of the limitations of the used scoring system. In addition, some studies reported different levels of HRQoL following different types of bariatric procedures [15].

In this study, a statistically significant association (p < 0.001) was found between the quality of life post-bariatric surgery and multiple sociodemographic factors, including age, gender, nationality, educational level, marital status, and history of chronic diseases.

In another study conducted in the same target population, many factors were found to be associated with increased quality of life after bariatric surgery, such as a significant reduction in BMI after the surgery and a higher preoperative reported score in self-esteem and HRQoL [26]. In general, obese patients tend to report a lower HRQoL compared to the general population, and the quality of life tends to normalize one year after successful bariatric surgery and lasts up to 10 years postoperatively [28].

In addition to the mentioned factors that are associated with quality of life after bariatric surgery, there are other important factors related to mental health that have been found to predict postoperative HRQoL. This can include personality, psychiatric disorders before the surgery, and improvement in depression symptoms [15].

## Conclusions

Quality of life after bariatric surgery was mostly reported to be fair or within the average range. Many sociodemographic factors were found to be associated with the reported quality of life, including age, gender, nationality, educational level, marital status, and history of chronic disease or comorbidities. Larger and better-designed studies are required to understand the different factors that may predict the quality of life following bariatric surgery emphasizing the different types of bariatric procedures and duration since surgery. A similar study including follow-up is strongly recommended as the outcome of any bariatric procedure should be strongly judged by the length of follow-up.
